# Our Summer at Maplewood. Chap. V. Griddle-Cakes and Other Things

**Published:** 1875-06

**Authors:** 


### CHAPTER V.

### GRIDDLE-CAKES AND OTHER THINGS.

"Kate, I wonder if we shall be permitted to go on in this quiet, restful way, all summer? I never enjoyed anything so much; but am all the while haunted by a fear that some prowling interloper will discover our retreat. Do you not think Alice wanders about too unguardedly, and is making too many acquaintances?"

I had barely time to respond "Nonsense, Cousin Emmeline," when the little silver bell tinkled a summons to breakfast. Leaving the roses ungathered, we went in, to find a vase of them already upon the breakfast-table, mingling their sweet odors with those of more substantial things.

When Alice, with a little air of pride, passed the cakes to me—such perfectly formed dainty looking things—the sight of them somehow called from their graves a spectral army of ghostly cakes, the cakes of other days, and of less careful cooks than our charming Alice. Haunted by their memory, I couldn't help saying to Emmeline, "What an abomination the griddle is in most houses, filling them with stifling atmospheres and unsavory smells. And yet this is not in the least degree a necessity. Cakes may be baked in the kitchen, and no one up-stairs be wiser or sadder therefor, if the cook knows how to tend the griddle. But if she floods it with grease which is allowed to burn before the cakes are placed upon it, not only is the house filled with smoke, but the cakes are blackened, and to a great extent spoiled, coming to the table with lacerated edges, bruised and torn, in their fierce conflict with fat and fire. Many cooks seem to take a special delight in causing the smoke of their torment, the griddle, to ascend unceasingly; and to this end they deluge it with grease, which, by the time it is filled with cakes, has been crowded close to the edge, where it hisses and bubbles, sputtering angrily all through the baking. Here and there on the griddle are drops of batter, impromptu little cakes undesigned by the cook, to which she pays no heed, unless, when lifting their more important kindred, she gives them an impatient push into the gutter of grease at the edge of the griddle, where they fry and burn uncared for. Sometimes it happens that these vagrants, black and dismal as they appear, are caught up accidentally and mixed with the cakes sent to the table. But oftener they are pushed off the griddle upon the range, where their illegitimate lives end in smoke."

"But, Kate, how is this nuisance to be prevented?"

"By using a suitable greaser in a proper manner. A good greaser may be made of pure lard placed just where it is required, by means of a linen swab, fastened to the end of a stick fashioned for the purpose; but the best greaser is a piece of fat pork either fresh or salted, two inches square and an inch thick, with the rind covering one of its broadest surfaces. Slip this piece of pork on a fork in such a manner as to bring the tines of the fork close to the rind, having the greaser below. With this, rub the griddle where the cakes are to be placed, wiping it well with a dry cloth after each baking. A griddle so treated will annoy no one, not even the cook, with smoke. Griddles and bread-pans should be used for nothing but the purpose for which they are specially designed, and should very rarely be washed with soap. If kept clean, they need little washing at any time, as a brisk rubbing with a dry cloth when they are warm, is all-sufficient, and keeps them in more perfect condition than frequent washing.

Buckwheat-cakes are perhaps more universally eaten in this country than any other kind, and at the same time are more invariably and unmitigatedly bad. A mean buckwheat-cake comes nearer my idea of total depravity in cakes than any other one. Cousin Emmeline, just imagine a Christian gentleman breakfasting on those tawny, leaden things,—leathery, gritty, sour, and half raw. In eating them, he forfeits a good share of self-respect, and goes his way after breakfast with a heavy heart in his bosom and a bitter taste in his mouth. If he did not know he was the victim of buckwheat-cakes he would be morally certain the whole world was against him, and his best friend had become his worst enemy.

A buckwheat-cake at its best is certainly the queen of the griddle, a very princess among cakes,—light, brown, crisp. It has no ragged edge, no tawny leaden-gray color, streaked with black. Sour? Why, the smoke of it is as grateful as sweet incense. You pat it tenderly with your knife, then spread it daintily with the sweet butter waiting to be gracious, adding syrup just to make the honeycomb illusion complete. I never knew a man after breakfasting on such cakes to contemplate suicide, or grumble if his wife asked him for "money."

Here Emmeline interrupted me with,—

"Kate, if I were you I would have Alice keep pencil and paper at hand and jot down such little impromptu speeches as this, on griddles and griddle-cakes, for your cook-book; for my opinion is that some of the best thoughts and choicest sayings of our fine writers never get into their books. They come and go, with the passing moment of inspiration, known only to the fortunate few who happen to be at table or in the drawing-room. But are you aware, Kate, that at the beginning of breakfast I asked you what kind of cakes these are that we are eating."

"Is it possible you don't know?"

"All things are possible, it seems, when you and Alice play your witcheries in the kitchen; but, although I'm not sure, I think these are rice-cakes. Rice-cakes are my favorite cakes, and these are certainly very nice." At this point Alice's musical laugh rippled into the conversation, ending with,—

"Rice-cakes, indeed, mamma; you ought to have seen the stale old bread that went to make up these little brown beauties."

Emmeline answered with indignation, not unmixed with disgust,—

"Bread-cakes, Alice, are my particular detestation,—soft, sticky, and disagreeable.

"Oh fie, mamma! how shocking! But I hasten to heap coals of fire on your head, by giving you the exact recipe for these delicate, delicious cakes. Pour over a pint of bread-crumbs, the same measure of boiling milk. If the milk has been skimmed, a small piece of butter must be used.

Cover closely, and let it stand over night. In the morning mash to a smooth paste, and beat thoroughly with it the yolks of two eggs. Then gradually add half a pint of cold milk, beating meanwhile, and half a pint of flour with which a measure of baking-powder has been sifted. Lastly, add the whites of the eggs, beaten to a stiff froth. These cakes require longer baking than batter-cakes, and should be baked of a small size, as they are tender and easily torn, and, when served, should be spread over the plate, and not piled one upon the other."

"Rice-cakes, Alice, may be made in the same manner as bread-cakes, substituting well-boiled rice for bread-crumbs; wheat batter-cakes are made in the same way, only using more flour. Measure for measure of milk and flour are the usual proportions, which must be varied at the discretion of the cook, according to circumstances. When many eggs are used, the batter may be made thinner than when few are used. More butter is also needed in the one case than in the other."

"But, Kate," said Emmeline, "I wish to know how those queenly buckwheat-cakes are made?"

"To a quart of warm water add a pound of buckwheat-flour, four ounces of fine corn-meal, a gill of yeast, and a saltspoonful of salt. Beat all well together, cover closely, and let it rise over night. In the morning, add a quarter of a small teaspoonful of soda, dissolved in a little hot water. Any mixture of sugar or syrup in the batter, to bring out a spurious or superficial browning, must be avoided as a delusion and a snare. If the cakes are light, and the griddle hot, you have nothing to fear.

"Kate, in that cook-book of yours, I hope you will devote one whole chapter to the consideration of corn-bread."

"Thank you, Cousin Emmeline, for the suggestion. Corn-bread being a Southern institution or specialty, is very much neglected by Northern and Eastern cooks; or, when made by them, is so "doctored," as a general rule, that a genuine lover of the article scarcely recognizes it in the vile mixture of grease, soda, eggs, milk, and sugar, thickened with poor corn-meal ground so fine as to be destitute of character or sweetness. Southern corn is as much superior to that grown at the North as a Hubbard squash is to a yellow pumpkin; but Southern corn, ground as fine as the meal that is generally used at the North, loses much of its excellence. Southern cooks undoubtedly excel in the art of making corn-bread of various kinds, among which the pone and hoe-cake used to take first rank. But since open fires, Dutch ovens, and bake-kettles, have been superseded by ranges and cooking-stoves, the pone has given place to corn-bread, and the hoe-cake to "dodgers," and other varieties, baked upon the griddle, or in the oven as convenience dictates. The pone, in the old time, was made of meal, warm water, yeast, and a little salt; and, after becoming light by fermentation, was baked,—a large loaf, in a Dutch oven, for five or six hours. The hoe-cake was still more simple, being corn-meal mixed with warm water and a little salt, into a stiff dough, fashioned by the hands into cakes an inch thick, and baked on a board, tilted before the fire, or in the negro cabins upon the hoe, which no doubt gave it the name. The dodger or johnny-cake is a legitimate descendent of the hoe-cake, and I think deserves precedence in the corn-bread ranks. To make it, take a quart of corn-meal, a teaspoonful of sugar, and a saltspoonful of salt. Scald with boiling water, leaving the paste so thick that when moulded into cakes in the hand it retains its form, or if placed upon the griddle with a spoon, will remain heaped and not spread into thin cakes. Put a piece of butter, half the size of a pea, just where the dodger is to be placed on the griddle, and, when melted, lay the cake upon it. In like manner fill the griddle. When brown, turn them over; but just before turning, place a small bit of butter on each cake, and when turned, press it gently in the center to flatten the rounded surface, and bring the cake close to the griddle at the edges. These cakes should be an inch thick; and they require half an hour to bake. The heat must, of course, be moderate, and the cakes may be turned several times if necessary. After being on the griddle ten minutes, having been turned and browned on both sides, they can be transferred to a pan and a hot oven for twenty or thirty minutes, to finish baking; or they can be baked all together in a hot oven, if desirable. When properly made, these are very delicious breakfast-cakes; and I do not wonder that one accustomed to them, turns in disgust from the messes often presented under the name of corn-bread, in which grease and soda bear so prominent a part that the stuff smells and tastes like soap.

"Corn-muffins are very fine, made in this way. To a quart of corn-meal add an ounce of butter, a teaspoonful of sugar, and a saltspoonful of salt. Scald with boiling water, pouring the water on slowly, and stirring the mixture until all the meal is moistened. When cool, add the yolks of three eggs, and beat well through the dough; then add cold, sweet milk, a small portion at a time, beating thoroughly, until the batter is of usual thickness. Beat the whites to a stiff froth, and add half at a time, alternating with a measure of baking-powder mixed with a small quantity of meal. Bake in muffin-rings, gem cups, or shallow pans, in a quick oven. Made a little thinner, this batter may be baked upon the griddle as thin griddle-cakes,—although the Southern griddle-cakes, or 'slappers,' as the colored people call them, are made somewhat different. I stood by Aunt Nancy, a colored woman, who made them deliciously, and saw just how she did it. She put a quart of meal into a bowl, and in the center made a nest or hole, into which she dropped a lump of lard as large as a hickory-nut, a pinch of salt, and a small teaspoonful of sugar. Then she poured boiling water on slowly, stirring the meal until all was moistened, when, pressing it compactly together in the bottom of the bowl, she 'lef' it to swell, honey,' while she brought out the griddle and greaser, and got things ready for the baking. This done, she returned to the cakes, and having ascertained that the dough was not too warm, she broke into it three eggs, stirring them briskly, with a spoonful or two of cold milk, through the dough. From time to time she added cold milk in small quantities, continuing the stirring until the batter was quite thin, when she announced,—

"'Dem's done ready, now, miss.'"

"'But you've forgotten the soda, Aunt Nancy,'" I said.

"'Forgot de sody,' she repeated, with infinite disgust, 'what for should I go spile dem slappers wid sody?'"

"In answer to my question why she didn't beat the eggs before she put them into the batter, she said emphatically, "Better dis yere way, honey. You take my 'vice, and don't go trying no 'speriments wid dese yere slappers.' I did, however, try the experiment of beating the eggs separately, after which I agreed with Aunt Nancy that they were better 'dis yere way.'

"This same 'slapper' batter, left a little stiffer, baked in the oven in shallow pans, cut in squares and served hot, is a very fine specimen of corn-bread, sometimes called Cape May Bread."

"Cousin Kate, do you know why the 'slappers' are better for having the eggs beaten only in and with the batter?"

"I think I do. But I don't mean to tell you, Alice, for the reason that I wish you to solve those riddles yourself, the reading of which requires only a little mild thinking."

"Listen to this," exclaimed Alice, excitedly, on coming home one evening, from a visit to Lizzie. Unfolding the Westfield *Democrat* she read to us the following:—

About Ghosts.—For some weeks it has been the current report that ghostly visitants have taken possession of the Douglas mansion, which is in charge of the gardener during the absence of the family, and hold private seances there, on which occasions they materialize or assume the forms they wore while tarrying in this mundane sphere in years gone by. Although we have but little faith in either the ghosts or the reports, many of our readers appear to be deeply interested in both, and wish us to give them all the information that can be obtained upon the subject. We have therefore detailed a special reporter, in whom we have the utmost confidence, to work up the case and give us the benefit of his investigations, and we hope shortly to lay before the readers of *The Democrat* a full account of the ghosts and their doings. Our reporter has been on duty several days, but has not as yet made much progress. The gardener, he says, "doesn't interview worth a cent," and he adds very emphatically, "the old fellow's wife is more secretive than a clam." He is busily engaged reading up on "Katy King," "Honto," and the numerous other spirits that are said to materialize, so as to be equal to the emergency, and fully prepared to tackle any sort of a ghost that may present itself. The facts he has gathered are very few and unsatisfactory, but we have high hopes of his giving us something racy in a short time, for he is willing to swear that he has had frequent glimpses of a creature not of the earth, earthy, flitting about the garden and veranda in the twilight, with hair floating about her shoulders, and dressed in the traditional white robes that ghosts are said to assume when they appear to mortal eyes.

